# Antibiotic prescribing for children with upper respiratory tract infection: a Finnish nationwide 7-year observational study

**DOI:** 10.1007/s00431-022-04512-w

**Published:** 2022-05-23

**Authors:** Matti Korppi, Paula Heikkilä, Sauli Palmu, Heini Huhtala, Péter Csonka

**Affiliations:** 1 Department of Pediatrics, Tampere University Hospital, and Center for Child, Adolescent and Maternal Health Research, Faculty of Medicine and Health Technology, Tampere University, Arvo Ylpönkatu 34 (ARVO B235), 33014 Tampere, Finland; 2 Faculty of Social Sciences, Tampere University, Tampere, Finland; 3 Terveystalo Healthcare, Tampere, Finland

**Keywords:** Primary health care, Upper respiratory tract infection, Common cold, Children, Antibiotics, Guideline concordance

## Abstract

Upper respiratory tract infection (URTI) is a self-limiting viral infection and should not be treated with antibiotics. The aim was to evaluate antibiotic prescriptions for children with uncomplicated URTI in a large nationwide private clinic network between 2014 and 2020. Special focus was given to macrolide prescriptions and costs. The data were obtained from the electronic health records (EHR) of the largest private healthcare company in Finland (with about 250,000 paediatric visits annually across the country). The collected variables included diagnoses, age, visit year, speciality of the doctor, and prescribed antibiotics. The number of uncomplicated URTIs in < 18-year-old children was 156,187 (53.0% in boys). The prescription rate of antibiotics decreased from 18.0% in 2014 to 8.8% in 2020, and that of macrolides from 6.1 to 1.7%. The costs decreased accordingly. Paediatricians prescribed antibiotics less often than general practitioners or ear, nose, and throat specialists.

*Conclusion*: Antibiotic prescriptions for uncomplicated URTIs, especially macrolides, decreased substantially during the 7-year surveillance period; however, 8.8% of children still received unnecessary antibiotics. To further reduce unwarranted antibiotic prescriptions, active interventions are needed that can be performed by applying the available EHR system.

**Table:** 

**What is Known:** *• Upper respiratory tract infection (URTI) is the most common infection in children. Uncomplicated URTI is a self-limiting viral infection, and antibiotic treatment is not warranted.*	
**What is New:** *• Almost 9% of children with uncomplicated URTIs still received unnecessary antibiotics. Paediatricians prescribed antibiotics less often than general practitioners or ear, nose, and throat specialists. To further reduce unwarranted antibiotic prescriptions, active interventions are needed that can be performed by applying the available EHR system.*

**Supplementary information:**

The online version contains supplementary material available at 10.1007/s00431-022-04512-w.

## Introduction

Upper respiratory tract infection (URTI) is the most common infection in children, with an average of six to eight URTI episodes a year at preschool age [1]. URTIs lead to more healthcare provider visits and missed days from day-care, school, and work than any other illnesses. In young children, the symptoms of URTI include nasal congestion, general irritability, eating problems, nausea, and sometimes fever [1]. Older children have milder and mainly local symptoms, and fever, if present, is usually low grade [1]. The common cold is a self-limiting viral infection, and there is no curative treatment [1].

Ear and sinus infections and pneumonia are bacterial complications of URTI in children. Approximately 70% of children suffer from acute otitis media before the age of 2 years [2]. Acute bacterial sinusitis should not be considered if symptoms have lasted less than 10 days. In typical cases, symptoms worsen after a period of transient improvement in that time frame. However, the exact incidence of acute sinusitis is unknown [3]. The annual occurrence of community-acquired pneumonia (CAP) in children is between 0.2 and 0.6% [4–6]. According to current recommendations, these bacterial complications require antibiotics. Meanwhile, URTIs without any complications should not be treated with antibiotics [7]. Instead, most guidelines suggest that treatment should focus on parental counselling and on relieving children’s symptoms until the illness passes [8, 9].

The task force set up by the European Paediatric Association concluded that the over-consumption of antibiotics in children with spontaneously recovering respiratory tract infections is one of the most important healthcare problems in children in Europe [10]. Less consumption of antibiotics and better targeted prescriptions within primary care are necessary for controlling the emergence of resistant bacteria and the stewardship of currently available antibiotics.

The aim of the present study was to evaluate antibiotic prescriptions for children with uncomplicated URTIs in a nationwide private outpatient clinic network in Finland. Special focus was paid to macrolide prescriptions. An additional aim was to estimate the cost of prescribed antibiotics, as well as variations in antibiotic prescriptions based on age, time, and the speciality of the doctor.

## Methods

The study was conducted using data from the electronic health records (EHR) of Terveystalo, the largest private healthcare company in Finland, with about 250,000 paediatric visits annually around the country. Terveystalo has more than 300 clinics in each of the 20 hospital districts in mainland Finland.

The study population consisted of children aged < 18 years who presented with an outpatient visit at Terveystalo from 1 January 2014 through 31 December 2020 and received an URTI diagnosis (International Classification of Diseases, ICD 10^th^ codes J00, J06, J06.80, J06.89, J06.9). The children were classified into four age groups: < 2 years, 2–4.9 years, 5–11.9 years, and 12–17.9 years. Patients with a concomitant bacterial diagnosis were excluded (Table S1). Prescribed antibiotics were identified by the Anatomical Therapeutic Chemical Classification system (Table S2).

DynamicHealth (TietoEVRY, Finland) is a tool utilised by Terveystalo for handling EHR data. The data for this analysis were collected and combined from records containing the required personal information, visit information, diagnoses, and medications as registered in DynamicHealth by the practitioner. Patients’ birth date information was derived from the EHR and used in calculating patients’ age upon visit from 2014 to 2020. The doctors were classified into paediatricians; general practitioners (GP); ear, nose, and throat specialists (ENT); and others. The patient data was actively managed in adherence with the European Union’s General Data Protection Regulation (GDPR) and the data security legislation of Finland.

Antibiotic prescriptions were identified from the EHR, with the antibiotic-related cost estimated by the 2021 price of each product in euros. The data included the name of the prescribed medicine, but detailed information on the dosage and size of the prescribed packets was missing. In Finland, the Pharmaceutical Pricing Board determines the national reference prices for medicinal products, such as antibiotics, that are reimbursable under the Health Insurance Act. Thus, these prices are the same in every pharmacy across the country; therefore, these reference prices were used for cost estimation in this study. The costs were estimated with the lowest price (usually meaning the smallest size of packet) and the highest price (largest size of packet) for each antibiotic. In this way, variations in the costs could be found and were comparable between the study years. The annual costs and costs per patient were estimated with the presumption that every prescribed antibiotic was purchased.

### Statistical analysis

All URTI visits of all cases during the study period were included in the analyses. Therefore, power analyses or formal sample size calculations were not performed. IBM SPSS Statistics for Windows, version 26 (IBM Corp, Armonk, NY, USA), was used in the data analysis. The age and gender of the child, speciality of the doctor, and visit year were included as co-variates in the multivariate logistic regression, and the results were expressed as odds ratios (ORs) and 95% confidence intervals (CIs). The statistical analyses focused on percentages, considering that respiratory infections were reduced by lockdown measures throughout 2020 [11, 12]. These measures followed the World Health Organization announcement on 11 March 2020 that COVID-19 was officially a pandemic.

### Ethics

This study was a quality assessment and development project. All data were coded, and patients were not contacted. According to Finnish law, approval from the Ethics Committee was not required, and informed consent was not needed from the subjects and/or their legal guardians. This study was carried out in accordance with national regulations and was approved by the chief medical officer of Terveystalo.

## Results

**Fig. 1:**
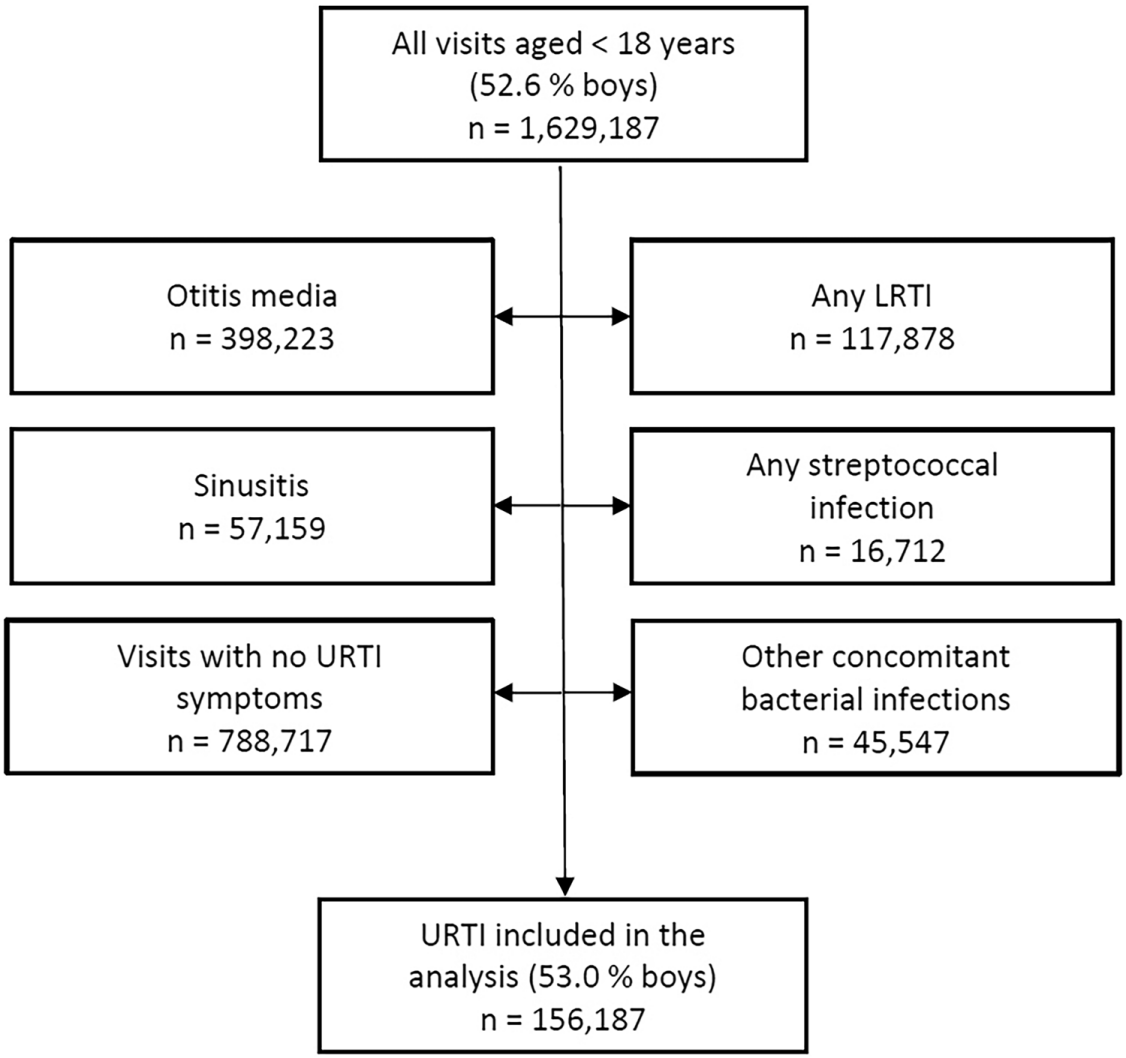
Flow chart of study inclusion and exclusion criteria for acute upper respiratory tract infection (URTI) visits during the period of 2014–2020. Side arrows indicate the exclusion criteria. N, number of visits; LRTI, lower respiratory tract infection

The total number of doctoral visits in < 18-year-old children was 1,629,187 (52.6% in boys) during the period of 2014–2020 (Fig. 1). After excluding pneumonia and other lower respiratory tract infections, bacterial complications, bacterial infections not associated with URTI, and visits without URTI symptoms, the number of pure URTI cases was 156,187 (53.0% in boys). The decrease in visits during 2020 was attributed to the COVID-19 pandemic (Table 1). The ages of the patients and treatments by speciality are shown in Table 1.

**Table 1:** The study population with upper respiratory tract infection (URTI)

	**URTI visits**	
	***n***	**%**
**All**	**156,187**	
**Year**		
2014	17,284	
2015	18,093	
2016	23,082	
2017	26,974	
2018	28,484	
2019	28,706	
2020	13,564	
**Age group (years)**		
< 2	34,844	22.3
2–4.99	43,459	27.8
5–11.99	45,672	29.2
12–17.99	32,212	20.6
**Speciality**		
Paediatrician	47,789	30.6
General practitioner	81,267	52.0
Ear, nose, and throat specialist	20,932	13.4
Other speciality	6,199	4.0

**Fig. 2:**
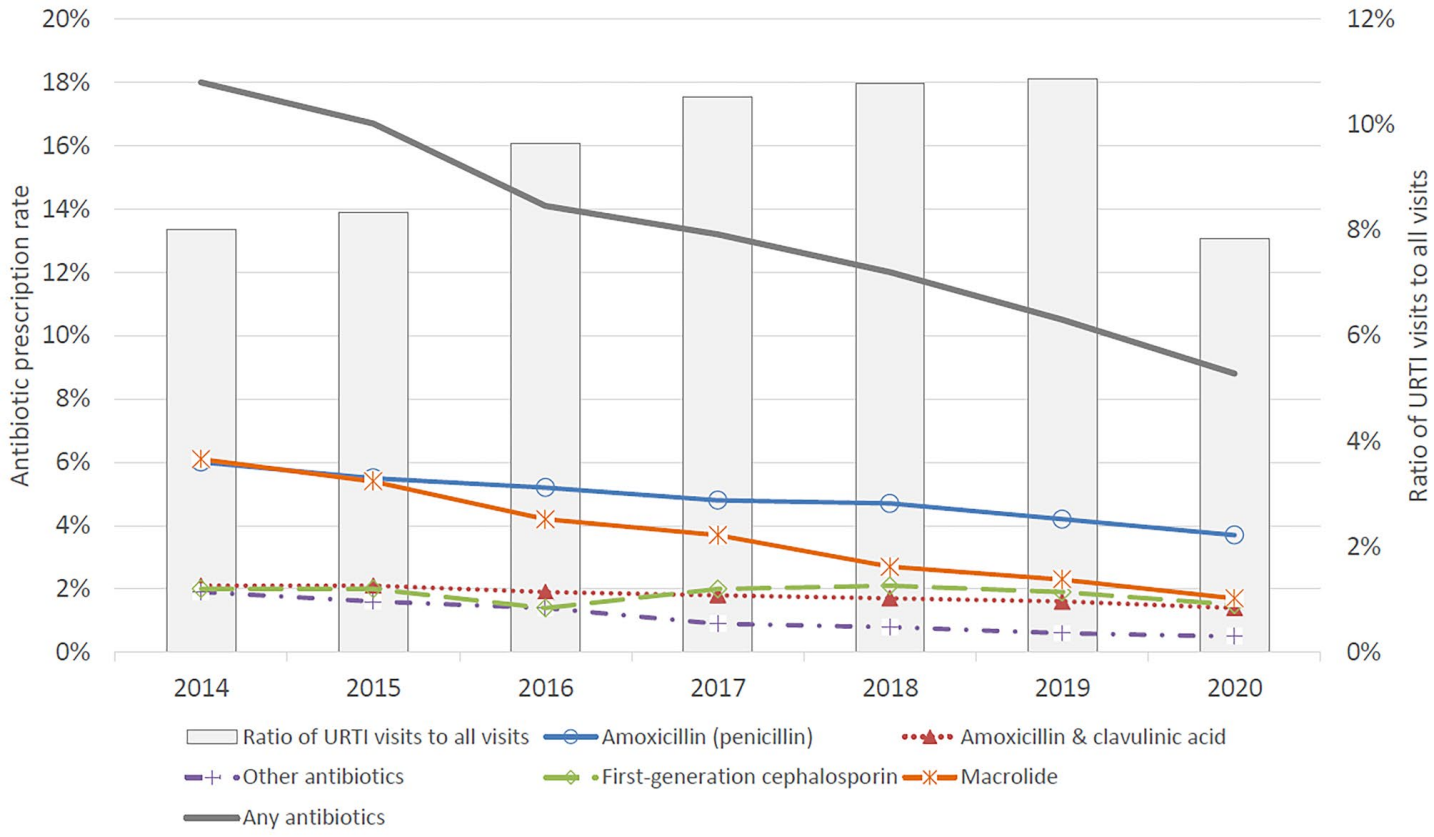
Antibiotic prescription rates for upper respiratory tract infection (URTI) [left axis] and URTI visit rates [right axis] yearly. Other antibiotics include pivmecillinam, ceftriaxone, trimethoprim, sulfamethoxazole/trimethoprim, sulfasalazine/trimethoprim, second-generation cephalosporin, and doxycycline. Total number of visits, 1,629,187. Number of URTI visits, 156,187

**Fig. 3:**
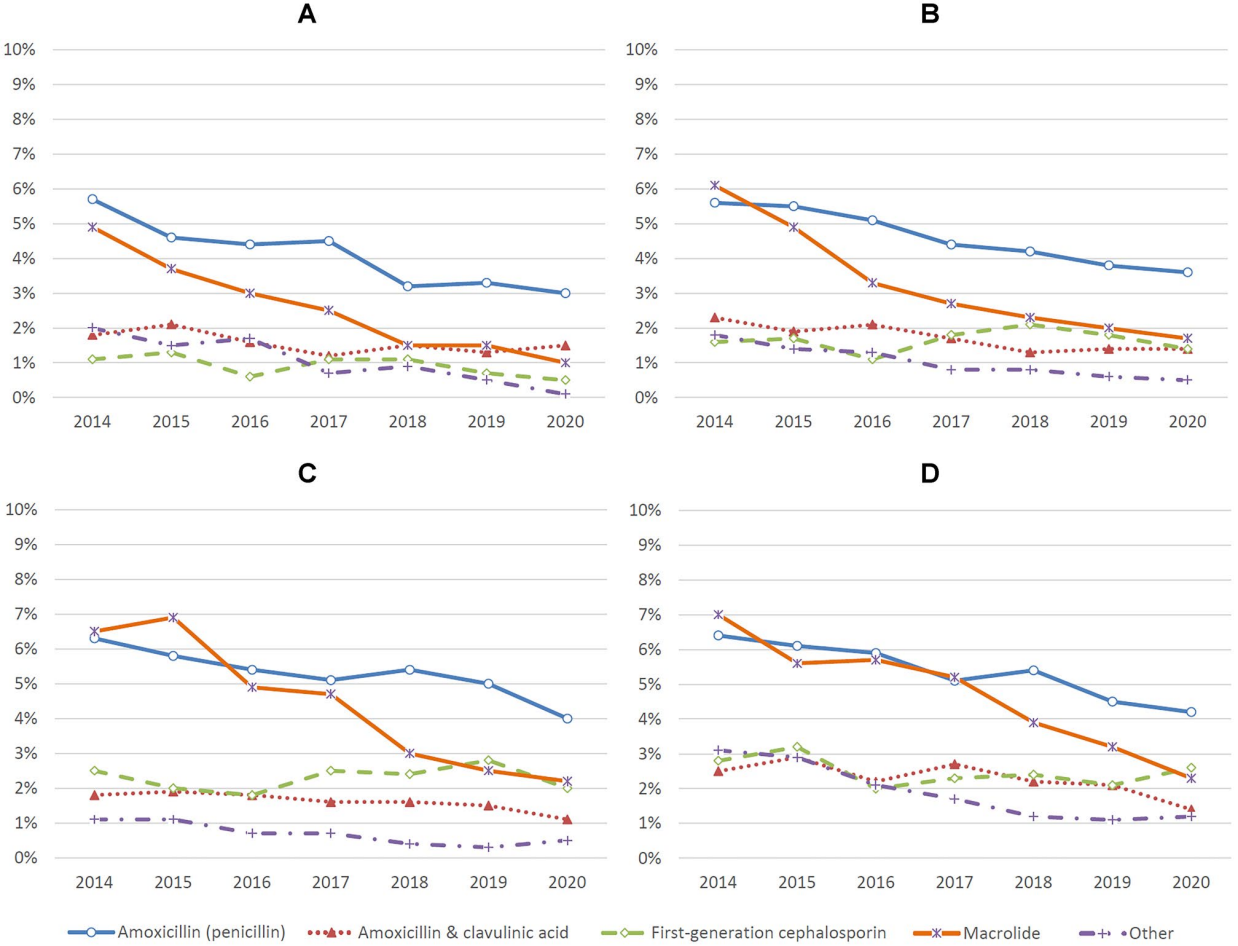
Antibiotic prescription rates for upper respiratory tract infection by age group. **A** Children < 2 years (*n* = 34,844). **B** Children 2–4.9 years (*n* = 43,459). **C** Children 5–11.9 years (*n* = 45,672). **D** Children 12–17.9 years (*n* = 31,212). Other antibiotics include pivmecillinam, ceftriaxone, trimethoprim, sulfamethoxazole/trimethoprim, sulfasalazine/trimethoprim, second-generation cephalosporin, and doxycycline

The overall prescription rate of antibiotics for URTI decreased constantly from 18.0% in 2014 to 8.8% in 2020 (Fig. 2). Specifically, this rate was 6.0% and 3.7% for amoxicillin and 6.1% and 1.7% for macrolides, respectively. In other words, prescriptions of antibiotics were halved, and those of macrolides decreased by 72.1% during the study period. The results were similar when the cases were analysed by age group (Fig. 3).

**Fig. 4:**
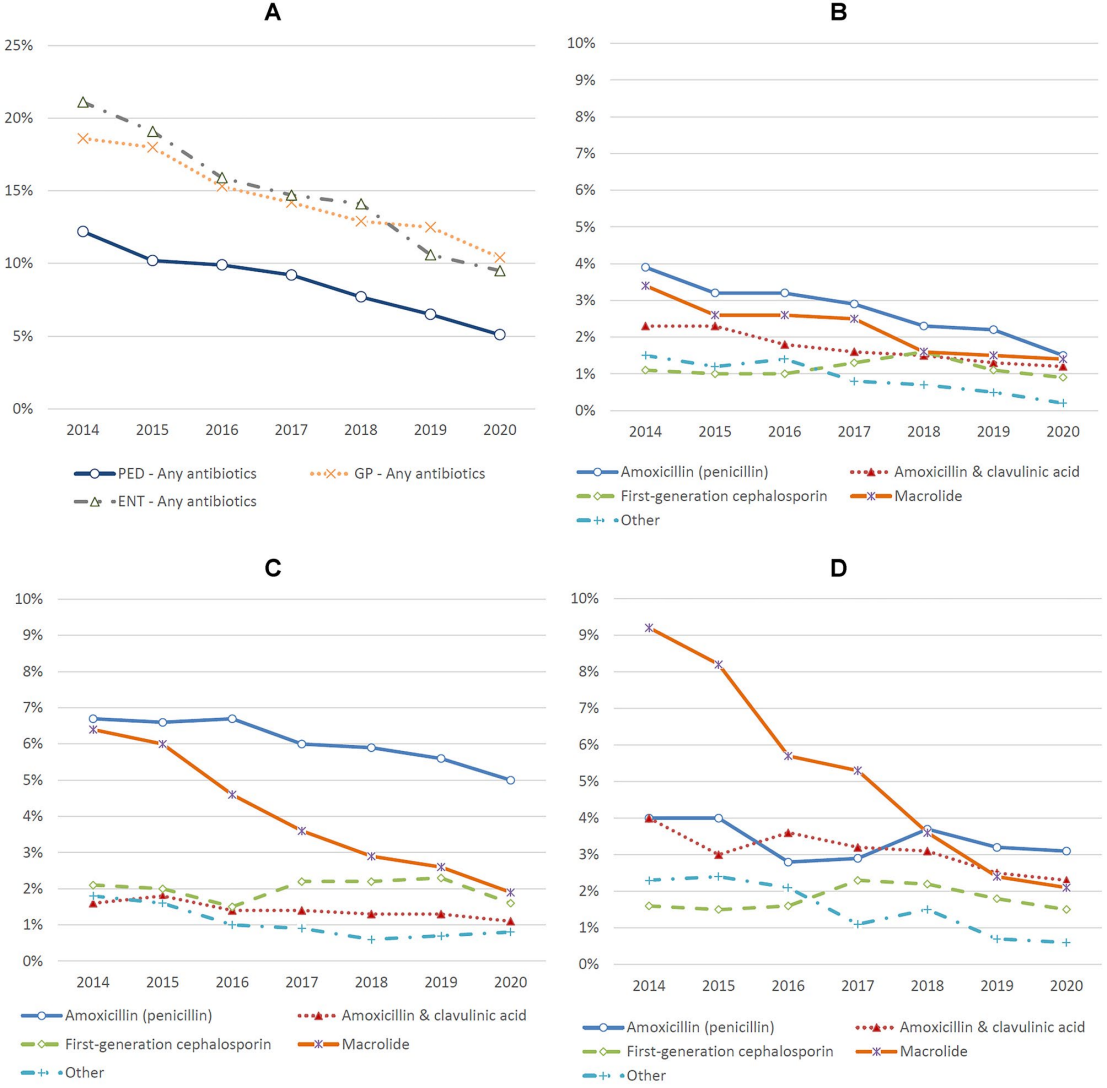
Antibiotic prescription rates for upper respiratory tract infection as diagnosed by specialists. **A** All patients (*n* = 156,187). **B** Patients treated by paediatricians (*n* = 47,789). **C** Patients treated by general practitioners (*n* = 81,267). **D** Patients treated by ear, nose, and throat specialists (*n* = 20,932). Other antibiotics include pivmecillinam, ceftriaxone, trimethoprim, sulfamethoxazole/trimethoprim, sulfasalazine/trimethoprim, second-generation cephalosporin, and doxycycline. PED, paediatrician. GP, general practitioner. ENT, ear, nose, and throat specialist

The prescription rate of antibiotics was the lowest among paediatricians in any year in 2020 at 5.1% (Fig. 4A). The respective figure for GPs was 10.4%, and for ENT doctors, it was 9.5%. Paediatricians reduced their prescriptions of all antibiotics, except those of first-generation cephalosporins (Fig. 4B). Amoxicillin was prescribed by GPs for 6.7% of children in 2014 and for 5.0% in 2020 (Fig. 4C). The respective figures for macrolides were 6.4% and 1.9%, illustrating a substantial decrease (Fig. 4C). The most notable change in prescription practice for ENT specialists was the dramatic (77.2%) decrease in macrolide prescriptions during the same time period (Fig. 4D).

Younger age (OR 0.97) and later visit year (OR 0.88) were associated with lower ORs for receiving antibiotics in a multivariate logistic regression adjusted by the age and gender of the child, visit year, and speciality of the doctor. Compared to paediatricians, the respective ORs were 1.64 for GPs and 1.71 for ENT specialists (Table S3).

**Fig. 5:**
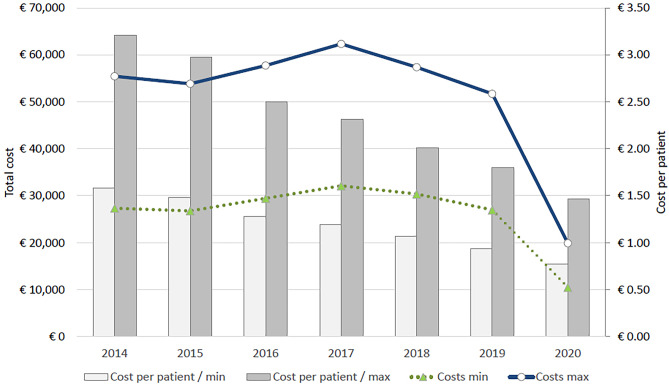
Cost of prescribed antibiotics per patient treated for upper respiratory tract infection. Total annual cost, left axis. Cost per patient, right axis. *n* = 156,187

In 2014–2017, the estimated annual total costs related to antibiotics followed a trend of the number of URTI visits. Meanwhile, in 2018 and 2019, URTI visits increased, but the annual total costs decreased remarkably, and in 2020, both the visits and costs decreased. The annual total costs varied between €62,327 in 2017 and €19,885 in 2020, when the highest antibiotic prices were used for estimation. The mean antibiotic-related cost per patient decreased consistently from €3.2 (in 2014) to €1.5 (in 2020) when the highest prices were used (Fig. 5); this demonstrated a decrease of 53.1%.

## Discussion

### Summary

There were three main results in this real-life register-based study on antibiotic prescriptions in a population of over 159,200 children with URTI from 2014 to 2020 in the largest private healthcare company in Finland with nationwide coverage. First, antibiotics were prescribed for 18.0% of the URTI children population in 2014 and for 8.8% in 2020. This decrease was substantial and desirable; however, because URTI without any complications is self-recovering and does not benefit from antimicrobial therapy, too many patients were still prescribed antibiotics. The estimated cost per patient decreased accordingly: 53.1% for all antibiotics. Second, a substantial decrease (72.1%) took place in macrolide prescriptions. This decrease was similar in all age groups and in all doctoral speciality groups. Third, paediatricians prescribed antibiotics less often than GPs and other specialists, a finding in agreement with other recent studies [13].

### Strengths and limitations

The main strength of the present study was that the analysed data came from outpatient clinics covering different areas across the country and consisted of almost 159,200 children younger than 18 years with uncomplicated URTI diagnoses. The information was electronically entered and obtained for this study from the centralised and uniformly coded EHR. Our study has some limitations. The data are retrospective and come from the private sector and thus may not fully represent the whole Finnish child population. Moreover, we lacked data on the lengths of the antibiotic treatments as well as on definite medicine purchases. It is also possible (though unlikely) that not all diagnosis codes were recorded during the URTI visit, and some children were treated (e.g., because of otitis media) without the code for a condition warranting antibiotic therapy being recorded.

The COVID-19 pandemic started at the end of 2019 and was ongoing during the last year of our study. Due to social distancing restrictions and hygiene recommendations, the circulation of all respiratory viruses was lower during than before the pandemic [11, 12]. In the present study, the number of doctor visits was less than half in 2020 when compared to 2017, 2018, or 2019. Decreased doctoral visits for URTIs may have further reduced the prescribing of antibiotics. However, the beneficial trend of less antibiotic prescribing was already seen before the pandemic. In addition, since the data are presented as ratios, it is unlikely that the change in visit numbers would have had a significant impact on the ongoing trends of less antibiotic prescribing from 2014 to 2020.

### Comparison with existing literature

A recent questionnaire study on parental antibiotic use for URTIs in their children included 3,188 families across three provinces in China [14]. 46.0% of parents gave their children with URTI antibiotics, with or without prescriptions. The possibility of buying antibiotics without a prescription is a problem in many low-to-middle-income countries [15]. In China, 70% of children with URTI symptoms visited a doctor, and 54.8% of them received antibiotics; 7.7% of the parents asked for and received a prescription [14]. In another study from China, the overall rate of antibacterial prescribing for children of < 5 years with URIs was on average low, but overuse of broad-spectrum cephalosporins and macrolides was an issue [16]. We agree with the conclusions of the Chinese authors that multifaceted interventions are necessary to solve the problem of self-medication with, over-prescription of, and parental demand for antibiotics.

In a French register study, the authors analysed 221,768 paediatric visits for different respiratory infections in a national sample, including 680 GPs and 70 community paediatricians [13]. The design of the study was similar to that of our present study. The antibiotic prescription rates of GPs and paediatricians were 21.7% and 11.6% for the common cold and 24.1% and 11.0% for other non-specified presumably viral respiratory infections, respectively. In addition, GPs seemed to prescribe broad-spectrum antibiotics more often than paediatricians did [13]. The figures in our study were lower—especially in 2020—but the difference between the GPs and paediatricians was evident. Likewise, paediatricians were more likely to adhere to guidelines for management of paediatric acute respiratory infections in a study from the USA [17].

A register study from Canada collected data from the national Electronic Medical Records Primary Care database [18]. The study included 341 physicians, 204,313 patients, and 499,570 encounters, and the prescriptions were classified based on the recorded diagnoses into indicated and non-indicated prescriptions. The overall rate of unnecessary antibiotic prescriptions was 17.6% for those < 2 years of age and 18.6% for those aged 2–18 years. In children, one-fourth of antibiotics were for conditions for which they are never indicated, such as the common cold [18]. The authors called for initiatives to reduce the use of antibiotics for the common cold and other presumably viral respiratory infections. In our present study, all children suffered from uncomplicated URTI; thus, most, if not all, of the antibiotic prescriptions were unnecessary.

The construction and dissemination of treatment guidelines have no doubt played a role in the change in clinical practices. Antibiotic stewardship programmes and other interventions have increased the awareness of doctors and parents about the indications, advantages, and disadvantages of antibiotics, the spread of antibiotic resistance in the population, and the need for activities to enhance antibiotic stewardship [19–22]. This information has been disseminated to doctors both through popular media to the public and through medical journals. Paediatric URTI plays a central role in these programmes, since URTI in children is so common, and uncomplicated cases, without exception, should not be treated with antibiotics [20]. Interventions to reduce overall antibiotic prescribing could be further enhanced through comprehensive EHR systems that allow real-time doctor-specific monitoring of prescriptions, construction of algorithms to guide clinical decision-making, and even an incorporation of steering tools on the platforms that doctors use in their day-to-day practice.

The decreasing trend of antibiotic prescriptions in children with URTI was constant during our study period. The current antibiotic consumption level, < 9% in 2020, has been reached by means of recommendations and information disseminated to doctors as well as by the increased general awareness regarding antibiotics among parents. The evidence-based Current Care Guidelines in Finland have a long history. For years, they have been published online and are easily accessible by doctors and laypeople free of charge. All guidelines emphasise that respiratory tract infections of viral origin should not be treated with antibiotics and that macrolides are not first-line alternatives for any respiratory infection, except for pertussis in infants.

The first Finnish Current Care Guidelines for lower respiratory tract infections in children, including CAP, pertussis, laryngitis, wheezing bronchitis, bronchiolitis, cough, otitis media, and sinusitis, were published in 2014. Macrolides are not recommended even for *Mycoplasma* CAP as monotherapy because *Streptococcus pneumoniae* is common in mixed infections and is not sufficiently susceptible to macrolides in Finland, like in many other countries [23, 24]. The Finnish Current Care Guidelines for otitis media were published in 1999 and thereafter regularly updated, lastly in 2017, recommend that the first-line antibiotics are amoxicillin or amoxicillin-clavulanic acid, and second-generation cephalosporins, trimethoprim-sulfa, and macrolides are the second-line drugs for those allergic to penicillin [25]. The Finnish Current Care Guidelines for sinusitis were published in 1999 and updated in 2013. These versions recommended amoxicillin or penicillin as a first-line therapy. In the latest 2018 update, amoxicillin-clavulanic acid became, together with amoxicillin, the first-line recommendation [26].

In addition to the readily available national guidelines in the healthcare company, where the present study was done, an active intervention was carried out during 2017–2020 aiming to stop prescribing cough medicines for children [27, 28]. The intervention included the release of information and educational training stressing the importance of refraining from antibiotics for self-limiting viral respiratory tract infections, such as the common cold. Many other intervention elements were part of the cough medicine programme, as discussed recently [27, 28]. To further decrease the use of antibiotics in children with URTI or other uncomplicated viral infections, more active interventions are needed to translate and incorporate the guidelines into everyday practice [29].

A study from the Netherlands evaluated the cost-effectiveness of GP- and parent-directed intervention aiming to reduce antibiotic prescriptions for respiratory tract infections in children. The intervention was effective, as 25% compared to 50% of the controls received an antibiotic over the study period. The mean cost per patient associated with a respiratory tract infection was higher in the intervention group (€217.95) compared to usual care (€207.68) from a societal perspective. However, the mean healthcare costs per patient, including the intervention costs, were lower (€45.72 vs. €50.38, respectively) [30]. In a Japanese study with 3,763,353 patients of < 20 years of age, a remarkable annual added cost of inappropriate antibiotic prescription for URTI was observed, varying from US$147.6 million in 2013 to US$103.9 million in 2016 [31]. This result is in line with our study; although the antibiotic-related cost per patient decreased during the study period, there were still extensive annual costs.

### Conclusions and implications for practice and research

Antibiotic prescriptions decreased constantly during the 7-year surveillance period of this study. Despite this, 8.8% of the children still received unnecessary antibiotics due to uncomplicated URTI cases. The most notable reduction in antibiotic use was for macrolides; this was seen in all age and speciality groups. To further reduce unwarranted antibiotic prescriptions, active interventions are needed, and such interventions should be, in the case of URTI, especially targeted to general practitioners and ear, nose, and throat doctors.

## Supplementary information

Below is the link to the electronic supplementary material.

Supplementary file1 (DOCX 105 KB)

## Data Availability

Anonymised data collected for the study and a data dictionary defining each field in the set will be made available 1 year after publication, following approval of a proposal and with a signed data access agreement. All data requests should be submitted to the corresponding author for consideration.

## Abbreviations

ATC: Anatomical Therapeutic Chemical classification system
CAP: Community-acquired pneumonia
CI: Confidence interval
EHR: Electronic health record
ENT: Ear, nose, and throat specialist
GP: General practitioner
ICD: International Classification of Diseases
PED: Paediatrician
OR: Odds ratio
URTI: Upper respiratory tract infection

## Declarations

### Transparency declarations

None to declare.

## References

[1] Heikkinen T, Järvinen A (2003). The common cold. Lancet.

[2] Armengol CE, Hendley JO, Winther B (2011). Occurrence of acute otitis media during colds in children younger than four years. Pediatr Infect Dis J.

[3] Revai K, Dobbs LA, Nair S (2007). Incidence of acute otitis media and sinusitis complicating upper respiratory tract infection: the effect of age. Pediatrics.

[4] Juvén T, Mertsola J, Waris M (2000). Etiology of community-acquired pneumonia in 254 hospitalized children. Pediatr Infect Dis J.

[5] Jain S, Williams DJ, Arnold SR (2015). Community-acquired pneumonia requiring hospitalization among U.S. children. N Engl J Med.

[6] Senstad AC, Surén P, Brauteset L (2009). Community-acquired pneumonia (CAP) in children in Oslo, Norway. Acta Paediatr.

[7] Shields MD, Bush A, Everard ML et al (2007) Recommendations for the assessment and management of cough in children. Thorax 63:iii1–iii15. 10.1136/thx.2007.077370 10.1136/thx.2007.077370 17905822

[8] Oikonomou ME, Gkentzi D, Karatza A et al (2021) Parental knowledge, attitude, and practices on antibiotic use for childhood upper respiratory tract infections during COVID-19 pandemic in Greece. Antibiotics 10. 10.3390/antibiotics10070802 10.3390/antibiotics10070802 PMC8300644 34356723

[9] Tan T, Little P, Stokes T (2008). Antibiotic prescribing for self limiting respiratory tract infections in primary care: summary of NICE guidance. BMJ.

[10] Størdal K, Wyder C, Trobisch A (2019). Overtesting and overtreatment—statement from the European Academy of Paediatrics (EAP). Eur J Pediatr.

[11] Isba R, Edge R, Auerbach M (2020). COVID-19. Pediatr Emerg Care.

[12] Kuitunen I, Artama M, Mäkelä L (2020). Effect of social distancing due to the COVID-19 pandemic on the incidence of viral respiratory tract infections in children in Finland during early 2020. Pediatr Infect Dis J.

[13] Trinh NTHH, Cohen R, Lemaitre M (2020). Community antibiotic prescribing for children in France from 2015 to 2017: a cross-sectional national study. J Antimicrob Chemother.

[14] Lin L, Harbarth S, Hargreaves JR (2021). Large-scale survey of parental antibiotic use for paediatric upper respiratory tract infections in China: implications for stewardship programmes and national policy: antibiotic use for paediatric upper respiratory tract infections in China. Int J Antimicrob Agents.

[15] Zawahir S, Lekamwasam S, Aslani P (2019). A cross-sectional national survey of community pharmacy staff: knowledge and antibiotic provision. PLoS ONE.

[16] Xue F, Xu B, Shen A, Shen K (2021) Antibiotic prescriptions for children younger than 5 years with acute upper respiratory infections in China: a retrospective nationwide claims database study. BMC Infect Dis 21. 10.1186/s12879-021-05997-w 10.1186/s12879-021-05997-w PMC8040226 33845771

[17] Frost HM, McLean HQ, Chow BDW (2018). Variability in antibiotic prescribing for upper respiratory illnesses by provider specialty. J Pediatr.

[18] Schwartz KL, Langford BJ, Daneman N (2020). Unnecessary antibiotic prescribing in a Canadian primary care setting: a descriptive analysis using routinely collected electronic medical record data. CMAJ Open.

[19] Choosing Wisely Canada: colds, flu, and other respiratory illnesses: don’t rush to antibiotics. https://choosingwiselycanada.org/colds-flu-respiratory-illnesses-dont-rush-antibiotics/. Accessed 15 Feb 2022

[20] Kenealy T, Arroll B (2013) Antibiotics for the common cold and acute purulent rhinitis SO-: Cochrane Database of Systematic Reviews YR-: 2013 NO-: 6. 10.1002/14651858.CD000247.pub3.www.cochranelibrary.com 10.1002/14651858.CD000247.pub3 PMC7044720 23733381

[21] Working group set up by the Finnish Medical Society Duodecim, The Finnish Paediatric Society and TFS of GP (2015) Treatment of lower respiratory tract infections in children - Finnish current care guidelines. https://www.kaypahoito.fi/hoi50098. Accessed 18 Sep 2019

[22] Kronman MP, Gerber JS, Grundmeier RW et al (2020) Reducing antibiotic prescribing in primary care for respiratory illness. Pediatrics 146. 10.1542/peds.2020-0038 10.1542/peds.2020-0038 PMC7461202 32747473

[23] Tapiainen T, Aittoniemi J, Immonen J (2016). Finnish guidelines for the treatment of laryngitis, wheezing bronchitis and bronchiolitis in children. Acta Paediatr.

[24] Tapiainen T, Aittoniemi J, Immonen J (2016). Finnish guidelines for the treatment of community-acquired pneumonia and pertussis in children. Acta Paediatr.

[25] The Finnish Medical Society Duodecim (2017) Otitis media (acute otitis media in children). The Current Care Guideline. www.kaypahoito.fi. Accessed 10 Mar 2022

[26] Sinusitis (2018) Current care guidelines. Working group set up by the Finnish Medical Society Duodecim and the Finnish Association of Otorhinolaryngology – Head and Neck Surgery. In: Helsinki: The Finnish Medical Society Duodecim. https://www.kaypahoito.fi/hoi38050. Accessed 10 Mar 2022

[27] Csonka P, Heikkilä P, Koskela S (2021). Cough and cold medicine prescription rates can be significantly reduced by active intervention. Eur J Pediatr.

[28] Palmu S, Heikkilä P, Kivistö JE (2022). Cough medicine prescriptions for children were significantly reduced by a systematic intervention that reinforced national recommendations. Acta Paediatr.

[29] Korppi M (2022). Antibiotic stewardship programmes had a low impact on prescribing for acute respiratory tract infections in children. Acta Paediatr.

[30] Dekker ARJ, van der Velden AW, Luijken J (2019). Cost-effectiveness analysis of a GP- and parent-directed intervention to reduce antibiotic prescribing for children with respiratory tract infections in primary care. J Antimicrob Chemother.

[31] Tsuzuki S, Kimura Y, Ishikane M et al (2020) Cost of inappropriate antimicrobial use for upper respiratory infection in Japan. BMC Health Serv Res 20 10.1186/s12913-020-5021-1 PMC7048145 32111202

