# Supplementary material for: Antibiotic prescribing for children with upper respiratory tract infection: a Finnish nationwide 7-year observational study
Source: Eur J Pediatr. 2022 May 23;181(8):2981–90. doi: 10.1007/s00431-022-04512-w (PMC9126572; doi:10.1007/s00431-022-04512-w)
Supplement: Supplementary file 1 — Supplementary file1 (DOCX 105 KB) [file 431_2022_4512_MOESM1_ESM.docx]

**Supplement material**

**Table S1.** Competing diagnoses codes. International Classification of Diseases 10.

| Appendicitis: K35.0, K35.1, K35.9, K36.  Bronchitis: J20, J20.0, J20.1, J20.2, J20.3, J20.4, J20.5, J20.6, J20.7, J20.8, J20.9, J21.0, J21.8, J21.9, J21.99, J22  Clamydia: A70, A71.0, A71.1, A71.9.  Croup: J05.0, J05.1  Cystitis: N30, N30.0, N30.9.  Influenza and viral pneumonia: J09, J10.0, J10.1, J10.8, J11, J11.0, J11.1, J11.8, J12, J12.0, J12.1, J12.18, J12.19, J12.2, J12.8, J12.9  Laryngitis: J04.0, J06.0  Mykobacterial infection: A31, A31.0, A31.9.  Otitis media: H65.0, H65.1, H65.2, H65.3, H65.4, H65.9, H66.0, H66.1, H66.2, H66.3, H66.4, H66.9, H67.0. Sinusitis: J01.0, J01.1, J01.2, J01.3, J01.4, J01.8, J01.9.  Pharyngeal infection: J02.0 J02.8, J02.9, J03.0, J03.8, J03.9, J39.0, J39.1.  Pneumonia: J13, J14, J15, J15.0, J15.1, J15.2, J15.3, J15.4, J15.5, J15.6, J15.7, J15.8, J15.9, J16, J16.0, J16.8, J18.9  Pyelonephritis: N10.  Sialoadenitis: K11.2, K11.3.  Skin infection: L00, L01, L01.0, L01.1, L02, L02.1, L02.2, L02.3, L02.4, L02.8, L02.9, L03, L03.0, L03.1, L03.2, L03.3, L05.0.  Streptococcal infections: J02.0, J03.0, J13, A49.1, B95.0, B95.1, B95.2, B95.3, B95.4, B95.5, B95.6, B95.7, B95.8, A38, A40.3, A46, I01.9 |
| --- |

**Table S2**. Antibiotic groups. Anatomical Therapeutic Chemical (ATC) classification system.

| Amoxicillin (penicillin)  J01CA04 Amoxicillin  J01CE02 Phenoxymethylpenicillin    Amoxicillin & clavulinic acid  J01CR02 Amoxicillin/Clavulanic acid    First-generation cephalosporin  J01DB01 Cefalexin    Macrolide  J01FA01 Erythromycin  J01FA06 Roxithromycin  J01FA09 Clarithromycin  J01FA10 Azithromycin  J01FA15 Telithromycin    Other  J01AA02 Doxycycline  J01DC02 Cefuroxime  J01CA08 Pivmecillinam  J01DD04 Ceftriaxone  J01EA01 Trimethoprim  J01EE01 Sulfamethoxazole/Trimethoprim  J01EE02 Sulfasalazine/Trimethoprim  J01MA01 Ofloxacin  J01MA02 Ciprofloxacin  J01MA06 Norfloxacin  J01MA12 Levofloxacin  J01XA01 Vancomycin  J01AA07 Tetracycline |
| --- |

**Table S3.** Use of antibiotics for upper respiratory tract infection. Multivariate logistic regression analysis.

|  | **OR** | **95% CI for OR** | | **p-value** |
| --- | --- | --- | --- | --- |
| **Variables in the equation** |  | **Lower** | **Upper** |  |
| Age | 0.97 | 0.97 | 0.98 | <0.001 |
| Gender (boy) | 1.02 | 0.99 | 1.05 | 0.178 |
| Visit year | 0.88 | 0.87 | 0.89 | <0.001 |
| Speciality |  |  |  |  |
| Paediatrician | 1.00 |  |  |  |
| General practitioner | 1.64 | 1.57 | 1.70 | <0.001 |
| Ear nose and throat specialist | 1.71 | 1.62 | 1.80 | <0.001 |
| Other speciality* | 3.67 | 3.43 | 3.91 | <0.001 |

*Only 4 % of the children were treated by other specialists. OR, odds ratio.
